# Cheating the CHA_2_DS_2_-VASc Score: Thromboembolism in Apical Hypertrophic Cardiomyopathy

**DOI:** 10.1155/2014/189895

**Published:** 2014-11-20

**Authors:** Robin A. P. Weir, Nicola MacKenzie, Colin J. Petrie

**Affiliations:** ^1^Cardiology Department, Hairmyres Hospital, East Kilbride, Glasgow G75 8RG, Scotland; ^2^Cardiology Department, Monklands Hospital, Airdrie, Scotland

## Abstract

Atrial fibrillation increases the risk of systemic thromboembolism in general and stroke in particular. Not all patients who develop atrial fibrillation are at significantly heightened risk of thromboembolic complications, however, with the development of risk scoring systems aiding clinicians in determining whether formal anticoagulation is mandated. The most commonly used contemporary scoring systems—CHADS_2_ and CHA_2_DS_2_-VASc—provide a reliable means of assessing stroke risk, but certain cardiac conditions are associated with an increased incidence of thromboembolism without impacting on these risk scores. Hypertrophic cardiomyopathy, with its apical variant, is such a condition. We present a case of a patient with apical hypertrophic cardiomyopathy and atrial fibrillation who suffered dire thromboembolic consequences despite a reassuringly low CHA_2_DS_2_-VASc score and suggest that this scoring system is modified to incorporate the thromboembolic risk inherent to certain cardiomyopathies irrespective of impairment of left ventricular systolic dysfunction or clinical heart failure.

## 1. Introduction

Contemporary scoring systems such as CHADS_2_ and CHA_2_DS_2_-VASc assist in prediction of the risk of systemic thromboembolism in patients with paroxysmal or persistent/permanent atrial fibrillation (AF) but fail to acknowledge the risk associated with certain cardiomyopathies leading to inappropriate undertreatment of at-risk patients.

## 2. Case Presentation

**Figure 1:**
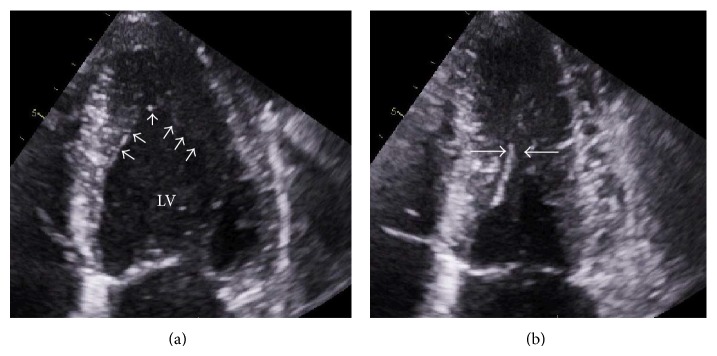
Apical hypertrophic cardiomyopathy. Transthoracic echocardiogram showing left ventricular (LV) apical hypertrophy in diastole ((a), arrows) and midcavity obliteration at end-systole ((b), arrows).

A 45-year-old male nonsmoker presented with a 2-hour history of acute pain in his left foot. His family doctor had detected 4 weeks previously at a routine annual health check that he was in AF and had arranged an echocardiogram which had shown marked hypertrophy of the apical left ventricular (LV) cavity (Figure 1(a), arrows), with obliteration of the midcavity in systole (Figure 1(b), arrows), appearances consistent with apical hypertrophic cardiomyopathy (HCM). There was no evidence of apical aneurysm formation, LV systolic function was within normal limits (LV ejection fraction (LVEF) calculated at 59% using Simpson's biplane method), and there were no valvular abnormalities. There was no other significant previous medical history, and anticoagulation had not been recommended due to his calculated CHA_2_DS_2_-VASc score of 0 [1].

**Figure 2:**
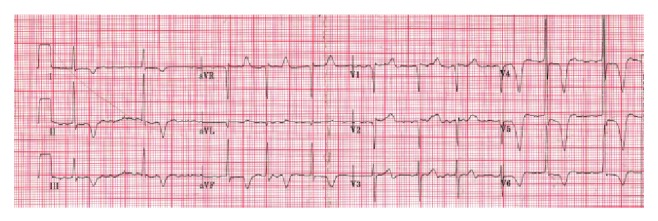
Preoperative ECG. Atrial fibrillation with voltage criteria for left ventricular hypertrophy and deep inferior/anterolateral T-wave inversion.

Physical examination on admission revealed a healthy but distressed patient with a pulse of 80 beats per minute irregularly irregular, blood pressure 150/57 mm Hg, and absent left lower limb pulses distal to the left femoral pulse. The left foot was pale and cold with capillary refill delayed at 5 seconds, although no signs of cutaneous necrosis were present. Vascular examination of the right lower limb was normal. There were no added heart sounds or murmurs and no signs of cardiac decompensation. A 12-lead electrocardiogram was recorded, which confirmed AF (ventricular rate 72 beats per minute) with widespread deep T-wave inversion (Figure 2). Within 4 hours of admission to hospital, the patient underwent uncomplicated surgical left femoral embolectomy which restored adequate left lower limb perfusion. The patient was anticoagulated with warfarin and made an uncomplicated recovery, completing 24 weeks of warfarin therapy prior to cessation of formal anticoagulation (largely at the request of the patient, who had had recurrent epistaxis while on warfarin). Unfortunately, he suffered a disabling stroke 10 weeks after stopping warfarin, with evidence of a right temporoparietal infarct on CT brain imaging. Doppler ultrasonography of the carotid arteries was normal, with the clinical impression that he had suffered a further cardioembolic event. Warfarin was reinstituted and recommended as lifelong therapy following the acute phase of the stroke.

## 3. Discussion

The fact that the risk of systemic embolism in patients with nonvalvular AF is higher in older age and in the presence of hypertension, diabetes, or a prior cerebrovascular event is well documented [2]. Warfarin reduces the incidence of stroke in high-risk patients with nonvalvular AF but determining which patients merit lifelong anticoagulation remains challenging [3]. The CHADS_2_ score—incorporating congestive heart failure (CHF), hypertension, age ≥ 75, diabetes, and prior stroke/transient ischemic attack (TIA)—has been superseded by the recent CHA_2_DS_2_-VASc score [1]. This latter score assigns a point each for CHF, hypertension, diabetes, prior vascular event, female sex, and age ≥ 65, with double points awarded for age ≥ 75 and/or a prior stroke/TIA. Annualized stroke rates range from 0% for CHA_2_DS_2_-VASc 0 to 15.2% for a score of 9 (the highest possible) [1].

Whilst a CHA_2_DS_2_-VASC score of 0 does not equate to zero risk of thromboembolism, the risk of ischaemic stroke in males with AF and a CHA_2_DS_2_-VASc score of 0 is not significantly higher than age and sex-matched controls in sinus rhythm [4]. Anticoagulation is usually recommended for CHA_2_DS_2_-VASc ≥1, which equates to a stroke risk of 1.3% per annum. Despite a diagnosis of apical HCM, the subject of this study had a CHA_2_DS_2_-VASC score of 0 at the outset, having never had clinical heart failure and with preserved LV systolic function. The apical variant is reported in up to ~7% of Western and ~25% of Asian patients with HCM [4, 5]. Long-term outcome studies have shown that apical HCM is not associated with increased cardiovascular mortality but heart failure, arrhythmias (particularly AF), and myocardial infarction are more common [6]. Studies of patients with AF and HCM (incorporating apical HCM) have reported a stroke rate of 3.75% per 100 patients per annum [7]. The mechanism by which apical HCM increases the risk of thromboembolism is unclear. Apical segmental dysfunction with consequent aneurysm formation, which has been observed to occur in the absence of epicardial coronary artery disease and is perhaps related to microvascular disease and reduced coronary flow reserve, is a potential mechanism, compounded by local eddying and stagnation of blood flow [8]. Left atrial enlargement is an independent risk factor for stroke in patients with AF; the management of the patient with HCM (apical or other) and left atrial enlargement but without documented atrial arrhythmia remains challenging, although at present prophylactic anticoagulation is not warranted. We recommend rigorous monitoring for atrial arrhythmias in such patients.

Failure to score a point on the CHA_2_DS_2_-VASc score at the time of diagnosis of this patient with AF and HCM reassured the attending physician that the risk of systemic embolism was sufficiently low to negate the requirement for formal anticoagulation. We caution physicians attending patients with AF and a cardiomyopathy without impairment of systolic function to determine the risk of systemic embolism for the individual patient and suggest that the current definition of the “C” in CHA_2_DS_2_-VASc—heart failure or moderate-to-severe LV systolic dysfunction (LVEF ≤ 40%)—should perhaps be modified with an acknowledgement of the risk that certain cardiomyopathies impose despite the absence of heart failure or significant systolic dysfunction [1].
